# Supplementary material for: Content-rich biological network constructed by mining PubMed abstracts
Source: BMC Bioinformatics. 2004 Oct 8;5:147. doi: 10.1186/1471-2105-5-147 (PMC528731; doi:10.1186/1471-2105-5-147)
Supplement: Additional File 2 — The original results of the above study (non-essential files are deleted to keep the file size under the limit set by BMC bioinformatics). [file 1471-2105-5-147-S2.bz2 › chilibotAdditionalFile2/dip30/751ID11872715E18134/html/FEPA_TONB.html]

 


 **FEPA** and **TONB** 
  
Found 47 abstracts in PubMed, retrieved 30.  
 

 What does Google say? 
 PDF only 
| .edu only 

---

**Interactive relationship** (e.g. stimulation, inhibition, etc)

**Neutral relationship**- Regions of Escherichia coli  **TonB**  and  **FepA**  proteins essential for in vivo physical interactions.  Ref: 9150216 J Bacteriol, 1997
- Protonmotive force, ExbB and ligand bound  **FepA**  drive conformational changes in  **TonB** .  Ref: 10209752 Mol Microbiol, 1999
- The Escherichia coli  **FepA**  protein is an energy and  **TonB**  dependent, ligand binding porin that functions as a receptor for the siderophore ferric enterobactin and colicins B and D.  Ref: 9268330 J Biol Chem, 1997
- The transport processes through  **FepA**  are energy dependent, relying on the periplasmic protein  **TonB**  to interact with  **FepA** .  Ref: 11532122 Mol Microbiol, 2001
- Through this interaction,  **TonB**  tranduces energy derived from the cytoplasmic membrane across the periplasmic space to  **FepA** .  Ref: 11532122 Mol Microbiol, 2001
- This possibility was supported by the finding that  **TonB**  carboxyl terminal truncations that retained Gln 160 were unable to participate in  **TonB**   **FepA**  complex formation, indicating that this site alone was not sufficient to support the physical interactions involved in energy transduction.  Ref: 9150216 J Bacteriol, 1997
- Characterization of in vitro interactions between a truncated  **TonB**  protein from Escherichia coli and the outer membrane receptors FhuA and  **FepA** .  Ref: 11292793 J Bacteriol, 2001
- The PCR mutagenesis strategy targeted the N terminus because it forms a plug inside the  **FepA**  barrel that is expected to be involved in ligand binding, ligand transport, and interaction with  **TonB** .  Ref: 11532122 Mol Microbiol, 2001
- The 84kDa Irp protein exhibits features including a  **TonB**  box and a 50 amino acid region that can adopt occluded beta barrel structures similar to the plug domain of the Escherichia coli FhuA and  **FepA**  crystal structures.  Ref: 11524163 Vet Microbiol, 2001
- Surprisingly,  **FepA**  with deletions of the globular domain amino acids 3 to 150 and 17 to 150 was previously reported to retain significant sensitivity to colicins B and D and to use ferric enterochelin, all in a  **TonB**  dependent fashion.  Ref: 12218040 J Bacteriol, 2002
- Crystal structures of two outer membrane transport proteins, FhuA and  **FepA** , and biochemical data reveal strong long range conformational changes of the proteins upon binding of Fe 3 siderophore complexes and in response to energy transfer from the cytoplasmic membrane into the outer membrane via the  **TonB**  ExbB ExbD protein complex.  Ref: 11934617 Curr Opin Microbiol, 2002
- The coupling of  **FepA**  L7 to OmpF, OmpC, or OmpA was  **TonB**  independent and was unaffected by the uncouplers CCCP carbonyl cyanide m chlorophenylhydrazone and DNP dinitrophenol but completely inhibited by cyanide.  Ref: 12169616 J Bacteriol, 2002

**Non-interactive relationship** (e.g. studied together, co-existance, homology, etc.)

- Under iron replete conditions,  **TonB**  and  **FepA**  were present at 335 78 and 504 165 copies per cell respectively.  Ref: 11967085 Mol Microbiol, 2002
- The application of biophysical methodologies to the  **TonB**  dependent porin,  **FepA** , ostensibly reveal the opening and closing of its channel during ligand uptake, a phenomenon that was predicted but not previously demonstrated.  Ref: 10066479 Curr Opin Microbiol, 1998
- In Escherichia coli, the primary players known in this process to date are  **FepA** , the  **TonB**  gated transporter for the siderophore enterochelin.  Ref: 11967085 Mol Microbiol, 2002
- We propose a plausible model of binding of  **TonB**  to FhuA and  **FepA** , two  **TonB**  dependent outer membrane receptors.  Ref: 11328822 J Biol Chem, 2001
- ExbB and ExbD each formed two unique cross linked complexes which were not dependent on the presence of  **TonB** , the outer membrane receptor protein  **FepA** , or the other Exb protein.  Ref: 9811664 J Bacteriol, 1998
- In this study, we report the per cell numbers of  **TonB** , ExbB, ExbD and  **FepA**  for cells grown under iron replete and iron limited conditions.  Ref: 11967085 Mol Microbiol, 2002
- To further understand  **TonB**  interaction with the beta barrel, in the present study, proteins with deletions of amino acids 1 to 15 to 15 0 to 152, and 17 to 150 in  **fepA**  were constructed and expressed in a deltafepA strain.  Ref: 12218040 J Bacteriol, 2002
- In the present study, a mutation in the  **TonB**  box of the ferric enterochelin receptor  **FepA**  resulted in the loss of detectable in vivo chemical cross linking between  **FepA**  and  **TonB** .  Ref: 9150216 J Bacteriol, 1997
- DALTV of FecA could be functionally replaced with the core  **TonB**  boxes of FhuA DTITV and  **FepA**  DTIVV.  Ref: 12618451 J Bacteriol, 2003
- The enterobactin degradation product, dihydroxybenzoylserine DBS was as effective as NE in stimulating the growth of E. coli and mutations in  **fepA**  or  **tonB**  abolished the DBS dependent growth stimulation.  Ref: 12379665 Infect Immun, 2002
- Although these receptors may vary in complexity and composition, the key protein involved in the transport of iron as iron, haem or iron siderophore across the outer membrane is a  **TonB**  dependent receptor with an overall structure presumably similar to that determined recently for Escherichia coli FhuA or  **FepA** .  Ref: 10383753 Mol Microbiol, 1999
- The crystallographic structures of two  **TonB**  dependent receptors FhuA and  **FepA**  have recently been determined.  Ref: 12409204 Biochim Biophys Acta, 2002
- Furthermore, the absence of  **FepA**  or indeed of all the iron responsive outer membrane receptors did not alter this association of  **TonB**  with the outer membrane.  Ref: 11872715 J Bacteriol, 2002
- H 6  **TonB**  captured both the ferrichrome iron receptor FhuA and the ferric enterobactin receptor  **FepA**  from detergent solubilized outer membranes in vitro.  Ref: 11292793 J Bacteriol, 2001
- We consider a variety of different colicins, including A, B, D, E1, E3, Ia, M and N, that penetrate through the porins OmpF,  **FepA** , BtuB, Cir and FhuA, to subsequently interact with a few targets in the periplasm, including TolA, TolB, TolC and  **TonB** .  Ref: 12423783 Biochimie< MedlineTA>Biochimie, 2001
- Although the presence of the siderophore enterochelin also known as enterobactin greatly enhanced detectable cross linking between  **TonB**  and the outer membrane receptor,  **FepA** , the absence of enterochelin did not prevent the localization of  **TonB**  to the outer membrane.  Ref: 11872715 J Bacteriol, 2002
- In contrast, the  **TonB**   **FepA**  ratio varied with iron status and according to the method used for iron limitation.  Ref: 11967085 Mol Microbiol, 2002
- They include the OmpA membrane domain, the OmpX protein, phospholipase A, general porins OmpF, PhoE, substrate specific porins LamB, ScrY and the  **TonB**  dependent iron siderophore transporters FhuA and  **FepA** .  Ref: 10931321 Mol Microbiol, 2000
- The plug domain of  **FepA** , a  **TonB**  dependent transport protein from Escherichia coli, binds its siderophore in the absence of the transmembrane barrel domain.  Ref: 11526207 Proc Natl Acad Sci U S A, 2001
- Amino acid replacements at one site in this region were found to affect energy transduction but did not appear to greatly alter  **TonB**  conformation or the formation of a  **TonB**   **FepA**  complex.  Ref: 9150216 J Bacteriol, 1997
- Quantification of known components of the Escherichia coli  **TonB**  energy transduction system  **TonB** , ExbB, ExbD and  **FepA** .  Ref: 11967085 Mol Microbiol, 2002
- The B. bronchiseptica  **tonB**  operon present on a plasmid did not complement an Escherichia coli  **tonB**  mutant, but inefficient reconstitution of enterobactin utilization was observed in one  **fepA**  mutant harbouring plasmid copies of the B. pertussis  **fepA**  homologue and  **tonB**  Bb operon.  Ref: 10517598 Microbiology, 1999
- Consistent with this hypothesis, E. coli strains with mutations in ferrienterobactin transport  **fepA**  or  **tonB**  or enterobactin biosynthesis entA did not respond to NE.  Ref: 12379665 Infect Immun, 2002
